# Targeted Cell Fusion Facilitates Stable Heterokaryon Generation *In Vitro* and *In Vivo*

**DOI:** 10.1371/journal.pone.0026381

**Published:** 2011-10-24

**Authors:** Michael A. Long, Fabio M. V. Rossi

**Affiliations:** The Biomedical Research Centre, University of British Columbia, Vancouver, British Columbia, Canada; University of Frankfurt - University Hospital Frankfurt, Germany

## Abstract

Induced cell fusion has enabled several important discoveries, including the phenomenon of nuclear reprogramming and may yet be applied as a novel therapy for degenerative diseases. However, existing fusogens lack the efficiency required to enable investigation of the epigenetic modifications underlying nuclear reprogramming and the specificity required for clinical application. Here we present a chimeric measles hemagglutinin, Hα7, which specifically and efficiently mediates the fusion of diverse cell types with skeletal muscle both *in vitro* and *in vivo*. When compared directly to polyethylene glycol, Hα7 consistently generated a substantial increase in heterokaryon yield and exhibited insignificant levels of toxicity. Moreover, this increased fusion efficiency enabled detection of chromatin modifications associated with nuclear reprogramming following Hα7-mediated fusion of human fibroblasts and mouse myotubes. Finally, Hα7 was also capable of increasing the contribution of transplanted fibroblasts to skeletal muscle repair *in vivo*, suggesting that this strategy could be used for therapeutic gene delivery.

## Introduction

Techniques for inducing the fusion of cells *in vitro* have been essential for research in a number of fields including the study of nuclear reprogramming [1], the production of monoclonal antibodies [2] and the generation of dendritic cell hybrids for cancer immunotherapy [3]. However, advances in these and other areas are currently encumbered by the limitations of traditional fusogenic agents. The most commonly utilized techniques for inducing cell fusion *in vitro*, namely polyethylene glycol (PEG) [4] and electrofusion [5] were first described roughly thirty years ago and although incremental refinements have gradually increased their efficacy, each of these methods remain notoriously inefficient. As a result of mechanisms that rely on random aggregation and membrane damage in order to achieve cell fusion, PEG and electrofusion protocols generally produce heterokaryons with low efficiency and high toxicity. Methods that employ micromanipulation [6], affinity crosslinking [7] or microfluidic devices [8] to properly pair two cell types are capable of increasing the efficiency of fusion. However, these systems continue to rely on the induction of membrane damage to initiate cell fusion.

*In vivo*, the ability of bone marrow derived cells and transplanted fibroblasts to contribute to the repair of several organs is largely thought to be due to the fusion of these cells with damaged tissues [9], [10]. This discovery has raised the prospect that cell fusion may represent a viable therapeutic strategy for several genetic and degenerative diseases. However, the inefficiency with which this phenomenon occurs has also precluded its therapeutic utility. Attempts to increase the efficiency of this process, including injection of snake venom toxins, appear to function by simply damaging tissue which in turn recruits inflammatory cells to the site of injury where they infrequently fuse to regenerating tissue [11]. Clearly, for such approaches to be clinically applicable, an increase in the specificity and efficiency of cell fusion is required.

Members of the *Paramyxoviridae* family of viruses, including measles and Sendai virus have long been known to induce cell fusion *in vivo* and *in vitro* [12], [13]. In the case of measles virus, infection is initiated via recognition of human CD46 or CD150 on the surface of cells by the viral hemagglutinin (H) protein [14], [15]. This interaction is believed to induce a conformational change in the associated viral fusion (F) protein, exposing a hydrophobic peptide, which inserts into the target plasma membrane and mediates fusion of the virus with the cell [16]. Subsequent display of measles H and F on the surface of infected cells then initiates fusion between neighboring cells, ultimately resulting in large multinucleated syncytia. Recently, a number of groups have altered the tropism of measles virus via addition of peptides [17], growth factors [18], single chain antibodies (scFv) [19] or cytokines [20] to the carboxyl-terminus of the hemagglutinin protein. The primary application of this technology has been the creation of oncolytic measles viruses, which are capable of specifically recognizing, infecting and killing tumor cells. However, considering that the H/F glycoprotein complex is capable of mediating cell fusion in the absence of viral infection [21], we hypothesized that chimeric measles hemagglutinin proteins could also be used to increase the efficiency of stable heterokaryon formation *in vitro* as well as for fusion-based cell therapy *in vivo*.

Here we present a targeted cell fusion approach based on a chimeric measles virus hemagglutinin glycoprotein, which is capable of generating stable heterokaryons with high efficiency both *in vitro* and *in vivo*. This modified measles virus hemagglutinin, Hα7, was produced by addition of a scFv that recognizes the muscle specific integrin, alpha7, to the carboxyl-terminus of a mutant hemagglutinin. Co-transfection of plasmids encoding Hα7 and measles F, induced fusion of all cell types tested with cultured skeletal muscle fibers. Moreover, the efficiency of Hα7-mediated fusion was clearly superior to PEG-mediated fusion and demonstrated insignificant levels of toxicity. Following Hα7-mediated fusion of human fibroblasts and mouse myotubes, transcription of the myogenic regulatory factors, MyoD and myogenin, as well as expression of neural cell adhesion molecule (NCAM) was activated in human nuclei. The level of human MyoD and myogenin mRNA detected following Hα7-mediated fusion was much greater than the level detected following PEG-mediated fusion, exceeding a ten-fold increase at most time points. Additionally, the increased efficiency of Hα7-mediated fusion enabled the detection of histone H3K9/K14 acetylation at the human MyoD promoter, demonstrating the utility of this method for the elucidation of epigenetic events underlying the process of reprogramming. Finally, transplanted fibroblasts expressing Hα7 specifically and efficiently fused with skeletal muscle fibers *in vivo*, suggesting that targeted cell fusion may represent a novel strategy for regenerative medicine.

## Results

### Design, construction and characterization of Hα7

**Figure 1:**
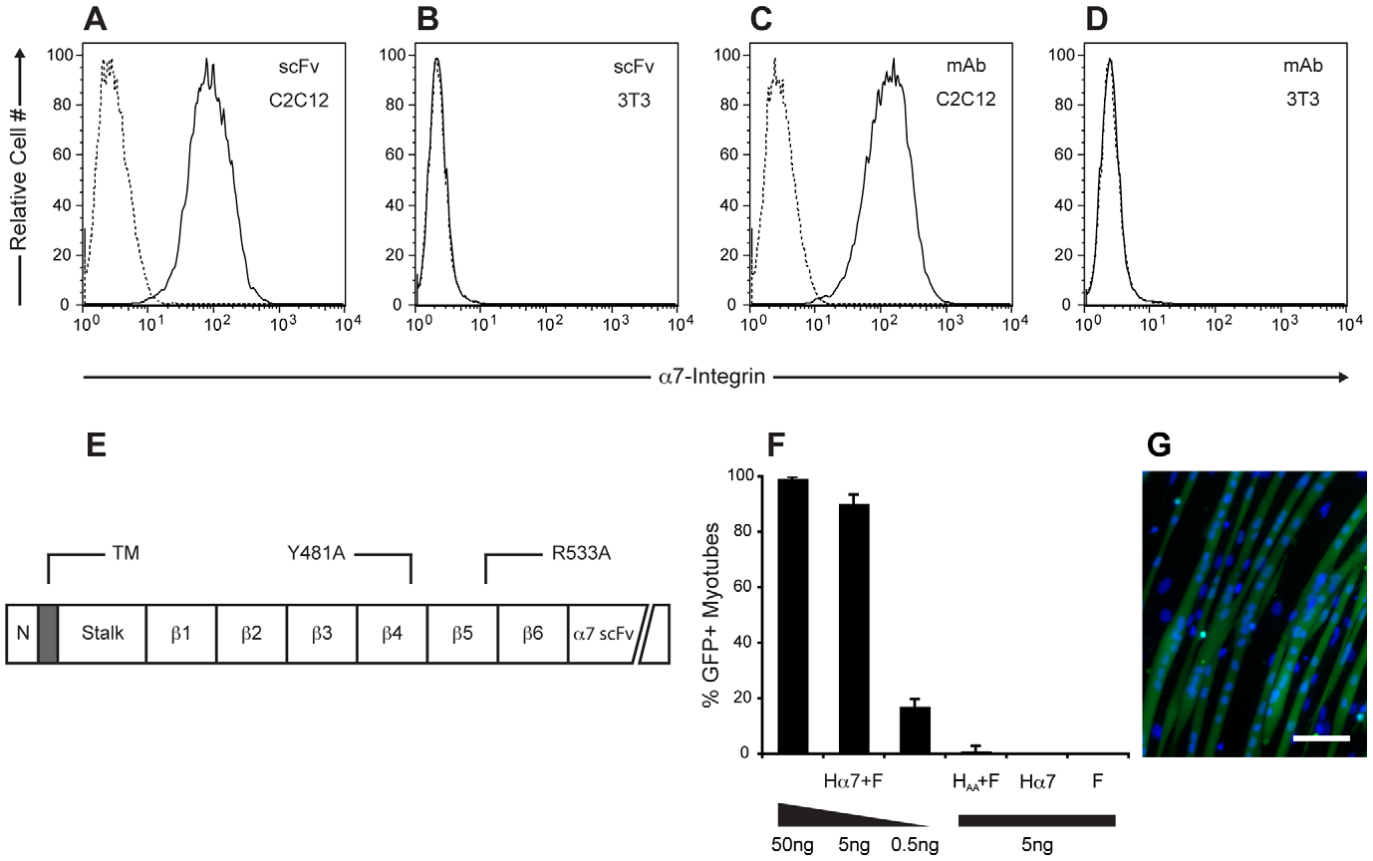
Design, construction and characterization of Hα7. (**A–D**) Evaluation of the anti-alpha7 integrin scFv by flow cytometry. The scFv (A, solid line) retains the ability of the parental monoclonal antibody (**C**, solid line) to stain C2C12 myoblasts, whereas neither antibody stains NIH/3T3 fibroblasts (**B** and **D**, solid line). In all plots, the staining level of cells incubated with secondary antibody alone is shown in dotted lines. (**E**) Schematic representation of Hα7, approximating the locations each blade (β1–β6) in the β-propeller fold as well as the location of mutations that abrogate CD46 binding (Y481A) and CD150 binding (R533A). The anti-alpha7 integrin scFv is displayed as a carboxy-terminal extension of the type II transmembrane glycoprotein. Standard one-letter abbreviations are used to denote amino acid residues. N: Amino-terminal cytoplasmic tail. TM: Transmembrane domain. (**F**) Hα7 mediates fusion of transfected 293T cells and differentiated C2C12 myotubes with an efficiency that is proportional to the amount of transfected plasmid and is dependent on the presence of the anti-alpha7 integrin scFv and the measles F protein. Data are shown as mean ± s.d. of three independent fusion experiments. (G) Morphology of myotubes following fusion. Scale bar, 100 µm

In order to generate a muscle-specific fusion reagent, we first constructed an anti-alpha7-integrin scFv from the well-characterized CA5.5 monoclonal antibody, which has been employed extensively in the purification and characterization of myoblasts [22]. As seen in Figure 1A–D, the scFv retains the specificity of the parental monoclonal antibody, demonstrated by its ability to stain C2C12 myoblasts but not NIH/3T3 fibroblasts. The anti-alpha7-integrin scFv was subsequently added to the carboxyl terminus of a mutated measles hemagglutinin, H_481A,533A_, which lacks the ability to bind either measles receptor [23] in order to create Hα7 (Figure 1E). We then tested the ability of cells exogenously expressing our chimeric hemagglutinin to fuse with differentiated skeletal myotubes. In order to accomplish this, 293T cells were transiently co-transfected with plasmids encoding Hα7, F and GFP. The following day, these cells were mixed with cultures of differentiated C2C12 myotubes and twenty-four hours after mixing, the percentage of GFP-positive myotubes was determined. As seen in Figure 1F,G transfection of as little as 5 ng of each plasmid was sufficient to induce fusion of 293T cells with the majority (90% +/− 3%) of myotubes in the culture. Importantly, very low numbers (1.0% +/− 2%) of GFP-positive myotubes were observed in the same assay when H_481A,533A_ was used in place of Hα7 and were completely absent when Hα7 or F were omitted from the transfection (Figure 1F).

### Verification of heterokaryon status following Hα7-mediated fusion

**Figure 2:**
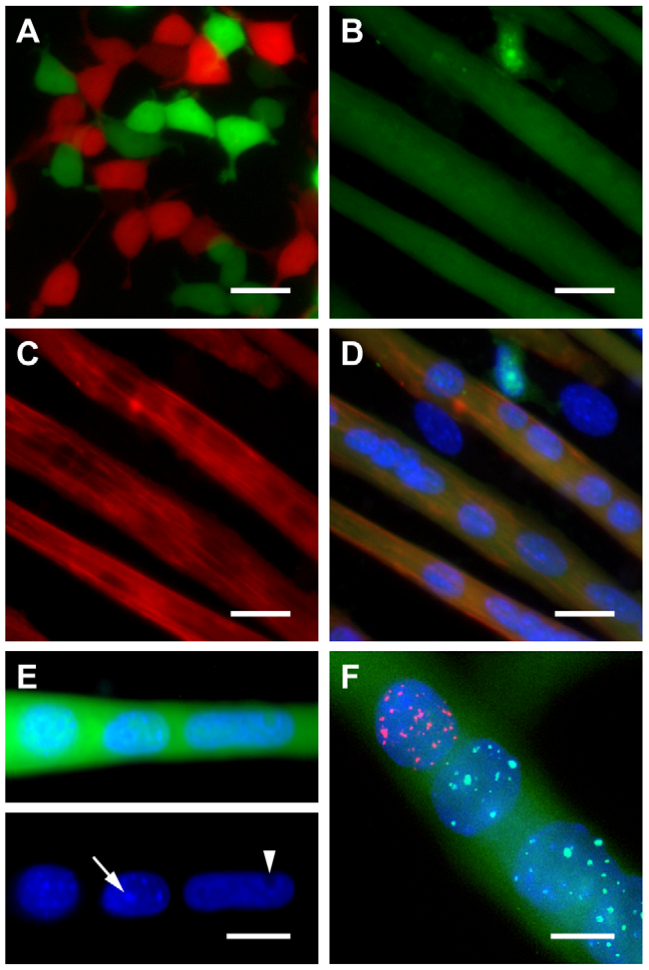
Verification of heterokaryon status following Hα7-mediated fusion. (**A**) Two populations of 293T cells labeled with either GFP or mCherry and co-transfected with Hα7 and F do not fuse with one another. (**B–D**) Following co-culture of transfected human 293T cells with differentiated mouse C2C12 myotubes, elongated GFP-positive cells (**B**) express sarcomeric myosin heavy-chain (**C**) and contain multiple nuclei (**D**, merged). (**E**) Differential DAPI staining demonstrates the presence of both human and mouse nuclei within heterokaryons. Mouse nuclei contain dense chromocenters (arrow) while human nuclei stain diffusely and exhibit dark nucleoli (arrowhead). (**F**) Fluorescent in situ hybridization of human α-satellite DNA (red) and mouse γ-satellite DNA (green) further confirms the presence of both human and mouse nuclei within heterokaryons. Scale bars, 25 µm (**A–D**), 20 µm (**E**), 10 µm (**F**)

In order to eliminate the possibility that the multinucleated, GFP-positive cells observed in co-cultures were exclusively derived from the homotypic fusion of transfected cells, we differentially labeled two populations of 293T cells via co-transfection of either GFP or mCherry in addition to Hα7 and F and subsequently co-cultured these cells in myogenic differentiation medium. This treatment did not result in the formation of syncytia (Figure 2A), suggesting that transfected 293T cells are unable to autonomously initiate the fusion process and demonstrating the inability of Hα7 to facilitate fusion between cells that do not express alpha7 integrin. In co-cultures of transfected 293T and C2C12 cells however, multinucleated, GFP-positive cells were found to express sarcomeric myosin heavy chain (Figure 2B–D), confirming the presence of proteins derived from both cell types within these syncytia. Furthermore, we identified the presence of both human and murine nuclei within these syncytia by differential DAPI staining (Figure 2E) as well as by fluorescent-in situ-hybridization (FISH) staining of human and murine satellite repeat DNA (Figure 2F). In the FISH assay, double-positive nuclei were never observed, indicating that following Hα7-mediated fusion of 293T cells with differentiated C2C12 myotubes, distinct nuclei are maintained within syncytia, thereby confirming the identity of these cells as true heterokaryons.

### Comparison of Hα7 and PEG induced fusion

**Figure 3:**
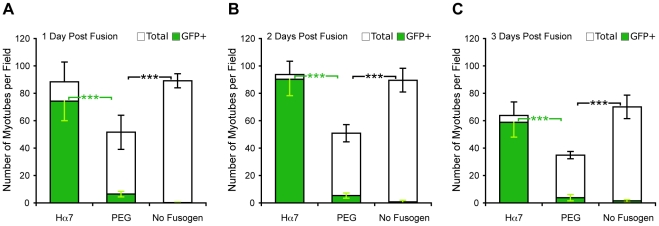
Comparison of Hα7 and PEG-mediated fusion efficiencies. (**A–C**) The total number of myotubes (white bars) as well as the number of GFP-positive myotubes (green bars) was determined by visual inspection of randomly selected, low power (5x) fields at 1 day (**A**), 2 days (**B**) and 3 days (**C**) post-fusion. The number of GFP-positive myotubes observed following Hα7-mediated fusion was significantly higher on all days than the number observed following PEG-mediated fusion (green asterisks). The total number of myotubes surviving PEG treatment was significantly lower than the total number observed in co-cultures lacking any fusogen (black asterisks). ***: p<0.001 (unpaired t-test). Data are shown as mean ± s.d. of three independent fusion experiments.

PEG remains the most widely used fusogenic agent for the production of heterokaryons. Therefore, we sought to compare the efficiency of Hα7-mediated fusion with that of a standard PEG-mediated fusion protocol. In this case, 293T cells were either co-transfected with plasmids encoding Hα7, F and GFP or transfected with a plasmid encoding GFP alone. The following day, equal numbers of 293T_Hα7/F/GFP_ or 293T_GFP_ cells were mixed with cultures of differentiating C2C12 cells and wells containing 293T_GFP_ were treated with PEG to induce fusion. The number of GFP-positive myotubes as well as the total number of myotubes per low-power field was determined daily thereafter for each condition. As expected, cells expressing Hα7 fused with the majority of myotubes in the culture (Figure 3A–C and Table S1). In cultures treated with PEG however, GFP-positive myotubes were much less frequent, with a maximum of 13% +/− 5% observed at twenty-four hours post-fusion (Figure 3A–C and Table S1). This finding is unlikely to be due to improper use of PEG, as previous studies employing this method have reported similar fusion efficiencies [24]. At all timepoints, the total number of myotubes surviving in the Hα7 treatment group was nearly twice as great as the number surviving PEG treatment. In fact, the total number of myotubes present following Hα7-mediated fusion was not significantly different from controls lacking any fusogen, demonstrating the lack of toxicity of this method. Ultimately, the combination of increased efficiency and reduced toxicity of Hα7-mediated fusion resulted in a 12 to 17-fold increase heterokaryon yield over the standard PEG-mediated fusion protocol (Table S2). A decrease in the total number of myotubes was observed on day three post-fusion as differentiated muscle cells began to contract and detach from the dish. However, this phenomenon uniformly affected the total number of myotubes across all treatment groups and did not preferentially affect GFP-positive myotubes within any group, suggesting that this is a normal behavior of myotubes in culture rather than an effect of fusion-inducing treatments.

### Nuclear reprogramming following Hα7-mediated fusion

**Figure 4:**
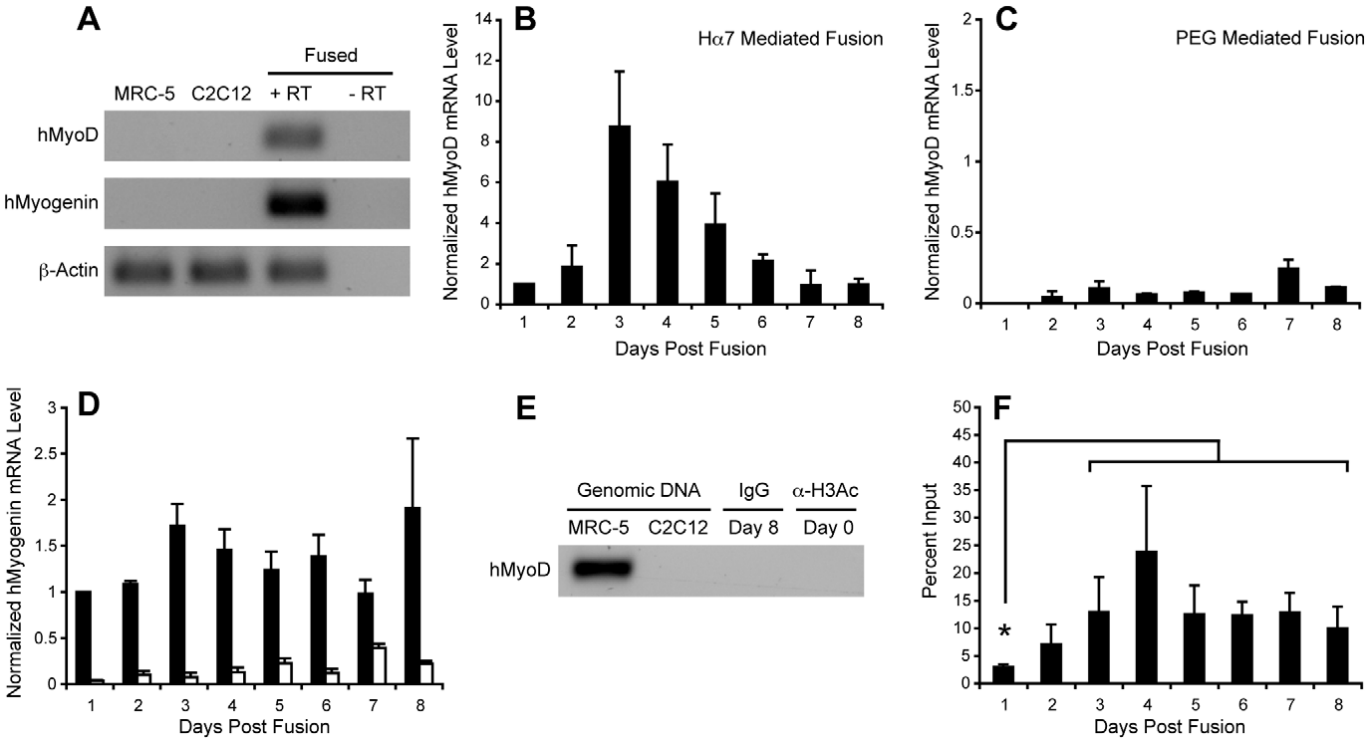
Nuclear reprogramming following Hα7 or PEG-mediated fusion. (**A**) Endpoint RT-PCR reactions demonstrating specificity of the primers used to amplify the human MyoD and myogenin transcripts. (**B–C**) Quantitative RT-PCR analysis reveals robust transcription of human MyoD following Hα7-mediated fusion (**B**) of MRC-5 cells and differentiating C2C12 myotubes, whereas the level of MyoD transcript observed following PEG-mediated fusion (**C**) is weak by comparison. (**D**) Likewise, the level of human myogenin transcript detected following Hα7-mediated fusion (black bars) was greater than the level detected following PEG-mediated fusion (white bars) at each timepoint. All quantitative RT-PCR values were normalized to β-actin transcript levels and subsequently to the mean expression level of Hα7-containing cultures on Day 1. (**E**) Endpoint PCR reactions demonstrating specificity of the primers used to amplify a segment of the human MyoD promoter (*lanes 1 and 2*), normal rabbit IgG chromatin immunoprecipitation (CHIP) control performed 8 days post-fusion (*lane 3*) and an αH3K9/K14 CHIP control performed on MRC-5 cells prior to fusion (*lane 4*). (**F**) Chromatin immunoprecipitation reveals early and stable induction of histone H3K9/K14 acetylation at the human MyoD promoter following Hα7-mediated fusion of MRC-5 cells and differentiating C2C12 myotubes. *: p<0.05 (unpaired t-test). All data are shown as mean ± s.d. of three independent fusion experiments.

A number of significant discoveries in the field of nuclear reprogramming have been made via fusion of various cell types with differentiating myotubes *in vitro* [1], [25]–[28]. However, the low efficiency of existing fusogenic agents has generally encumbered these experiments, slowing advances in our understanding of this phenomenon. Therefore, in order to demonstrate that the increased yield of heterokaryons generated via Hα7-mediated fusion is capable of overcoming these limitations, we analyzed induction of the human myogenic regulatory factor, MyoD, in heterokaryons comprised of MRC-5 human lung fibroblasts and differentiating C2C12 myotubes. As seen in Figure 4A, isolated MRC-5 cells do not express this transcription factor. However, following Hα7-mediated fusion, expression of human MyoD was rapidly upregulated, becoming detectable twenty-four hours after fusion and reaching a peak forty-eight hours later (Figure 4B). Transcription of human MyoD was then downregulated over time, resembling its kinetics of expression during the differentiation of normal myogenic cells [29]. In contrast, following PEG-mediated fusion of MRC-5 cells and differentiating C2C12 myotubes, expression of human MyoD was not detected until forty-eight hours after fusion and remained at low levels throughout the time course (Figure 4C). When compared directly, these data reveal that the level of human MyoD expression detected at daily intervals following Hα7-mediated fusion was up to 94-fold higher than the level observed following PEG-mediated fusion (Table S3).

In order to confirm that nuclear reprogramming following Hα7-mediated fusion is not a transient phenomenon, restricted to the expression of human MyoD, we also analyzed induction of a second myogenic regulatory factor, myogenin, in heterokaryons generated via Hα7 and PEG mediated fusion. As seen in Figure 4D, this transcription factor is rapidly induced and stably transcribed in heterokaryons generated via either protocol. However, the level of human myogenin transcript detected at daily intervals following Hα7-mediated fusion was up to 31-fold higher than the level observed following PEG-mediated fusion (Table S3). Finally, as further evidence of the extent and stability of nuclear reprogramming following Hα7-mediated fusion, we also detected expression of human NCAM in 85% +/− 9% of heterokaryons on day eight post-fusion (Figure S1).

Hα7-mediated fusion also enabled us to investigate the dynamics of histone H3K9/K14 acetylation at the human MyoD promoter during the reprogramming process. Although this modification is well known to be associated with transcriptional activation, its induction has not previously been described at individual loci during the process of reprogramming due to the insufficient yield of heterokaryons generated by PEG mediated fusion [30]. As seen in Figure 4E, histone H3K9/K14 acetylation of the human MyoD promoter is not detected in unfused MRC-5 cells, consistent with the fact that MyoD is not expressed in these cells. However, following Hα7-mediated fusion, histone H3K9/K14 acetylation of the human MyoD promoter is observed within twenty-four hours (Figure 4F). Although acetylation appears to peak at four days post-fusion, there is no statistically significant difference between any time point past day one, suggesting that histone H3K9/K14 acetylation of the human MyoD promoter reaches stable levels rapidly following fusion.

### Hα7-mediated fusion *in vivo*

**Figure 5:**
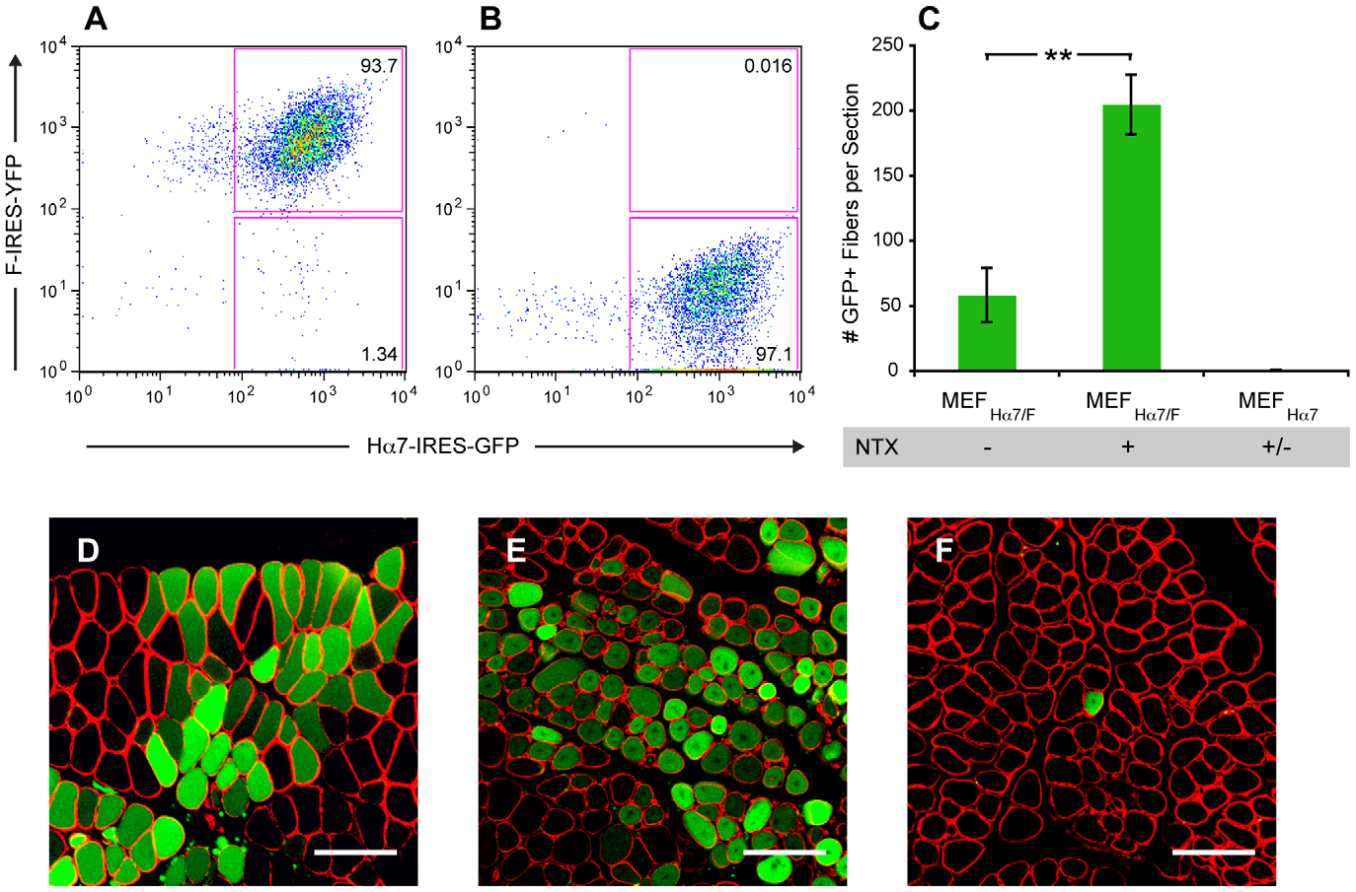
Hα7-mediated fusion *in vivo*. (**A,B**) Mouse embryonic fibroblasts infected with lentiviruses encoding Hα7-IRES-GFP and F-IRES-YFP (**A**) or Hα7-IRES-GFP alone (**B**) were purified by flow cytometry and transplanted intramuscularly into wild-type recipients. (**C**) Cells expressing Hα7 and F (MEF_Hα7/F_) efficiently fused with host myofibers and this process was greatly augmented by co-injection of notexin. Conversely, cells expressing Hα7 alone (MEF_Hα7_) did not fuse efficiently with host myofibers regardless of the presence or absence of notexin. Data are shown as mean ± s.d. **: p<0.01 (unpaired t-test). (**D–F**) Representative images demonstrating GFP-positive fibers (green) surrounded by a laminin sheath (red) in an undamaged recipient of MEF_Hα7/F_ cells (**D**), a notexin-damaged recipient of MEF_Hα7/F_ cells (**E**) and a notexin-damaged recipient of MEF_Hα7_ cells (**F**). Scale bar, 100 µm.

Finally, to evaluate the potential utility of targeted cell fusion for regenerative medicine, we investigated the ability of Hα7 to increase the efficiency of fusion between non-myogenic cells and skeletal muscle fibers *in vivo*. In order to accomplish this, mouse embryonic fibroblasts (MEF) were infected with the lentiviral vectors, LV-HIG and LV-FIY, which encode Hα7-IRES-GFP and F-IRES-YFP respectively, and cells were subsequently purified by flow cytometry (Figure 5A,B **)**. Doubly infected MEF_Hα7/F_ as well as singly infected MEF_Hα7_ control cells were then transplanted into the tibialis anterior muscle of C57BL/6 recipient mice. One week after transplantation, mice were sacrificed and hind limbs were examined for the presence of GFP-positive muscle fibers. As seen in Figure 5C,D, a large number of GFP-positive fibers exhibiting normal morphology and surrounded by basal lamina were observed in all recipients (n = 3) of MEF_Hα7/F_ cells.

Considering the fact that various forms of muscle damage are known to augment the contribution of transplanted cells to myofiber regeneration, we also transplanted MEF_Hα7/F_ and control MEF_Hα7_ cells via intramuscular co-injection with the myotoxic phospholipase, notexin [11], [31], [32]. As seen in Figure 5C, the number of GFP-positive fibers observed following co-administration of notexin was roughly 3.5-fold greater than the number observed in undamaged recipients. Although the GFP-positive fibers observed in notexin treated recipients were smaller than those observed in undamaged recipients (Figure 5D,E) this phenomenon is a common hallmark of regenerating muscle [33].

Importantly, among all recipients (n = 6) of MEF_Hα7_ control cells, only a single GFP-positive fiber was detected (Figure 5C,F). Furthermore, no GFP-positive cells were observed in the spleen, lung or liver following transplantation of MEF_Hα7/F_ into either damaged or undamaged recipients of (n = 6) (Figure S2).

## Discussion

We have created a cell fusion reagent, Hα7, which overcomes the low efficiency, high toxicity and lack of specificity exhibited by existing chemical and physical fusogens. As opposed to PEG and electrofusion, our system is based on a specific ligand-receptor interaction, which simultaneously promotes the proper pairing and efficient fusion of cells. This feature maximizes the generation of heterokaryons and virtually eliminates the non-productive formation of homokaryons. *In vitro*, cells expressing Hα7 routinely fused with over 90% of cultured myotubes and consistently yielded 12 to 17-fold more heterokaryons than a standard PEG-mediated protocol. A comparable increase in fusion efficiency has recently been described utilizing a microfluidic device to control cell pairing [8]. While this represents a significant improvement over existing techniques, the microfluidic device is limited to the manipulation of a maximum of six thousand cell pairs per run. Our method on the other hand, has no inherent limitations of scale and is therefore capable of producing far more heterokaryons per experiment.

The ability of Hα7 to increase fusion efficiency was not gained at the expense of cell viability. At all time points analyzed, the total number of myotubes present following Hα7-mediated fusion was not significantly different from controls lacking any fusogen. Moreover, heterokaryons generated via Hα7 treatment exhibited normal healthy morphology, characteristic of differentiated C2C12 myotubes. In contrast, PEG-mediated fusion resulted in the death of roughly half of the myotubes in culture. This difference is likely due to the fact that unlike PEG, the measles fusion glycoprotein complex is capable of initiating and stabilizing the fusion process without relying on the induction of membrane damage.

In heterokaryons generated via Hα7-mediated fusion of human fibroblasts and mouse myotubes, transcription of the myogenic regulatory factors, MyoD and myogenin, was activated in human nuclei. The level of human MyoD and myogenin transcripts detected following Hα7-mediated fusion was much greater than the level detected following PEG-mediated fusion, suggesting that this method may exhibit an increased sensitivity in general. While it remains possible that the expression of Hα7 and F may alter gene expression in heterokaryons, it is unlikely that this effect will be greater than the perturbations caused by the toxic effects of PEG. In fact, the use of a cell line such as MRC-5_ Hα7/F_, may provide a standardized reagent for studies of nuclear reprogramming and reduce inter-experimental variation caused by variable batches or usage of PEG.

Chromatin modifications are well-known to enhance or repress transcription and are likely to play a crucial role in establishing the gene expression networks required for nuclear reprogramming [34]. However, dynamic changes in chromatin modifications during the process of nuclear reprogramming remain largely undescribed due to the paucity of cells generated by traditional heterokaryon or transcription factor-based reprogramming methods. Here, we have exploited the greatly enhanced efficiency of Hα7-mediated fusion to demonstrate that acetylation of histone H3K9/K14 at the human MyoD promoter is rapidly induced following fusion. Moreover, once established, this modification remains stable despite the fact that expression of the MyoD gene is only transiently upregulated, suggesting that other epigenetic modifications may play a more important role in fine-tuning MyoD expression following fusion. Overall, these results demonstrate that the yield of heterokaryons generated by Hα7-mediated fusion is sufficient to enable identification of specific chromatin modifications at loci of interest. Therefore, we anticipate that this technology will facilitate the systematic identification of the factors and mechanisms involved in the process of nuclear reprogramming.

Skeletal muscle is naturally repaired by satellite cells, which proliferate and fuse to multinucleated myofibers [35]. However, in several human skeletal myopathies, ongoing cycles of fiber degeneration progressively deplete satellite cell numbers, impairing the ability of myofibers to regenerate by incorporation of new nuclei [36]. Unfortunately, satellite cells cannot be efficiently replaced by transplantation [37]. Therefore, we utilized Hα7-mediated fusion to create a novel method of delivering exogenous nuclei to myofibers. In these proof of principle experiments, transplanted MEF_ Hα7/F_ cells fused efficiently with recipient muscle fibers. The expression of donor derived GFP in these muscle fibers demonstrates the potential utility of Hα7-mediated fusion for the delivery of therapeutic transgenes to skeletal muscle. Although we have examined this reagent in the context of cell transplantation, Hα7-mediated targeting of non-cellular delivery vehicles may also be possible [38]. In addition, our results demonstrate that Hα7 is capable of enhancing fusion of fibroblasts and skeletal muscle fibers in an immunocompetent host. In agreement, Iankov *et al*, have demonstrated that cells infected with measles virus are capable of undergoing fusion *in vivo* even in the presence of pre-existing humoral immunity [39]. Therefore we further anticipate that Hα7-mediated fusion may facilitate the development of novel cell and gene therapies for skeletal myopathies.

While the syncytial nature of myofibers may render skeletal muscle innately amenable to a cell fusion based therapy, a small number of other therapeutically interesting cell types including cardiomyocytes and hepatocytes also naturally exist in multinucleated states [40], [41]. Furthermore, these cell types as well as others including Purkinje neurons and renal proximal tubule epithelial cells are known to tolerate fusion and exist as heterokaryons following bone marrow transplantation [9], [42]. However, as in the case of skeletal muscle, these fusion events are extremely infrequent and therapeutic effects are only observed in rare cases where heterokaryons exhibit a growth advantage over resident cells [42], [43]. Therefore, in order to treat the vast majority of pathologies in which positive selection does not occur, the efficiency of the fusion process must be increased. Clearly cell surface markers of suitable specificity must be validated for each cell type. However, if such markers can be identified, targeted cell fusion may represent a novel therapeutic approach to a number of degenerative diseases.

## Materials and Methods

### Ethics Statement

All experiments and procedures were approved by the Committee on Animal Care (Protocol #A09-0364) at the University of British Columbia, in accordance with the requirements of the Canadian Council on Animal Care (CCAC).

### Construction of Hα7

RNA was prepared from the CA5.5 hybridoma (RNeasy, Qiagen) and cDNA was produced utilizing Superscript II (Invitrogen) and an oligo-dT primer. The variable region of the immunoglobulin heavy chain was then amplified utilizing the primers CA5.5H-F: 5′-AAAAGATCTGGCCCAGCCGGCCCAGGTGCAGCTGAAGGAGTC-3′and CA5.5H-R: 5′-GACGGTGACCATGACTCCTTGG-3′. The variable region of the immunoglobulin light chain was amplified utilizing the primers CA5.5L-F: 5′-AAAGAGCTCGCTGACCCAGTCTCCTGCTTTG-3′ and CA5.5L-R: 5′-AAACTCGAGCGGCCGCCCGTTTCAATTCCAGCTTGGTGC-3′. A complete scFv was then assembled by cloning the heavy chain fragment upstream and the light chain fragment downstream of a glycine-serine (G_4_S_1_)_3_ linker contained in pASK85-9E10 utilizing BglII, BstEII and SacI, XhoI sites respectively, thereby creating pCA5.5scFv.

The CA5.5scFv was subsequently fused to a human light chain constant region by subcloning into pLC-huCκ with BglII and NotI. This plasmid, pCA5.5scFv- huCκ, was transiently transfected into 293T cells via standard calcium phosphate precipitation and forty-eight hours later, neat supernatant containing the CA5.5scFv-huCκ fusion protein was utilized to stain C2C12 myoblasts and NIH/3T3 fibroblasts in parallel with a 0.5 µg/mL dilution of the CA5.5 monoclonal antibody. The goat anti-human-kappa-PE (Southern Biotech) and goat anti-rat-PE (Southern Biotech) secondary antibodies were used to detect CA5.5scFv-huCκ and CA5.5 staining respectively. All flow cytometry data was collected with a Becton-Dickinson FACSCalibur and analyzed with FlowJo software. Following confirmation of specificity, the CA5.5scFv was fused to the carboxyl-terminus of a mutant measles hemagglutinin contained in pTNH6-H_AA_ using SfiI and NotI, thereby creating pHα7.

### *In vitro* fusion assays

293T and C2C12 cells were maintained in DMEM (Gibco) supplemented with 10% and 20% fetal bovine serum (Gibco) respectively. To induce differentiation, C2C12 cells were plated in DMEM supplemented with 2% horse serum (Invitrogen) on collagen-coated dishes (Sigma, Becton Dickinson) at a density of 4×10^4^ cells/cm^2^. Twenty-four hours later, cytosine β-D-arabinofuranoside (Ara-C) (Sigma) was added to a concentration of 1×10^−5^ M in order to eliminate proliferating myoblasts. 293T cells were transfected with calcium phosphate twenty-four hours prior to co-culture and were plated onto C2C12 cells at a density of 4×10^4^ cells/cm^2^. Co-cultures were initiated following two or five days of C2C12 differentiation and are referred to as differentiating or differentiated cultures respectively.

PEG-mediated fusion of cells was carried out as described previously [27]. Briefly, 293T cells were mixed with differentiating C2C12 myoblasts and allowed to settle and adhere for four to six hours. Medium was then completely aspirated and replaced with prewarmed 50% PEG 1500 (Roche) for sixty seconds. PEG was then removed and cells were washed three times in prewarmed DMEM. Cultures were subsequently maintained in DMEM supplemented with 2% horse serum, 1×10^−5^ M Ara-C and 1×10^−5^ M ouabbain (Sigma) to eliminate unfused human cells. Fusion efficiency was quantified at selected intervals by enumerating the total number of myotubes as well as the number of GFP-positive myotubes present in at least three randomly selected low power (5x) fields.

### Immunofluorescence and FISH

To detect myosin-heavy chain expression, heterokaryons were first fixed in 4% paraformaldehyde (PFA) for five minutes at room temperature, washed in PBS and permeabilized in 0.5% Triton X-100 for five minutes at room temperature. Cells were then stained with mouse anti-myosin-heavy chain (Developmental Studies Hybridoma Bank) overnight at 4°C, followed by a one hour incubation with goat anti-mouse Alexa 568 (Molecular Probes) at room temperature. Nuclei were counterstained with 4′,6-diamidino-2-phenylindole (DAPI) (1 µg/mL). To detect NCAM expression, live cells were incubated with a 1∶25 dilution of 5.1H11 hybridoma supernatant (Developmental Studies Hybridoma Bank) for one hour at 37°C, washed in differentiation medium and stained with goat anti-mouse Alexa 568 (Molecular Probes) for one hour at 37°C. Cells were then washed in differentiation medium, fixed with 2% PFA for ten minutes at room temperature, washed in PBS and permeabilized in 0.3% Triton X-100 for five minutes at room temperature. Nuclei were counterstained with 4′,6-diamidino-2-phenylindole (DAPI) (1 µg/mL). For FISH, cells were post-fixed with 4% formaldehyde, treated with 1 mg/mL pepsin, dehydrated in increasing series of ethanol and air-dried. Cells were denatured for three minutes at 80°C in hybridization mixture (70% formamide, 0.5 µg/mL of Cy-3–conjugated PNA probe specific to human α-satellite sequences (CTCCAAATATCCACTTGC), 0.5 µg/mL of Cy-5–conjugated PNA probe specific to mouse major satellite (GAAGGACCTGGAATATGG) and 0.25% (w/v) blocking reagent (DuPont) in 10 mM Tris (pH 7)). Hybridization was performed at room temperature for one hour and slides were then washed with 70% formamide/10 mM Tris (pH 7.2; twice for fifteen minutes each) and with 0.05 M Tris/0.15 M NaCl (pH 7.2) containing 0.05% Tween-20 (three times for five minutes each). Slides were dehydrated, air dried and counterstained with DAPI (0.2 µg/mL), and mounted in antifading solution (DABCO).

### Lentiviral Vectors

The transfer vector, pLV-HIG, was constructed by inserting the Hα7 cDNA contained in pHα7, downstream of the EF1α promoter in the third generation lentiviral vector, pCCL.sin.cPPT.EF1α.SET7.IRES.GFP.WPRE utilizing BamH1. The transfer vector, pLV-FIY, was constructed by first inserting the measles fusion protein cDNA contained in pCGF, downstream of the EF1α promoter in the same third generation lentiviral vector described above, utilizing Xma1 and Xba1. The EYFP cDNA contained in pEYFP (Clontech) was then cloned downstream of the IRES utilizing Nco1 and BsrG1.

Lentiviruses were produced by cotransfecting 293T cells with the appropriate transfer vector as well as with the packaging plasmids, pMDL, pRev and pVSVG utilizing calcium phosphate. Supernatant was harvested thirty-six to sixty hours later, filtered through a 0.45 µm filter (Pall) and transferred to subconfluent cultures of MRC-5 fibroblasts or p53^−/−^ C57BL/6 mouse embryonic fibroblasts in the presence of 5 µg/mL polybrene. Forty-eight hours after infection, cells were sorted (Becton-Dickinson FACSVantage) based on expression of YFP and/or GFP.

### Quantitative real-time gene expression analysis

Heterokaryons were generated as described above. Following fusion, RNA was harvested daily (RNeasy, Qiagen) from a single well for each treatment condition for a total of eight days. Purified RNA was treated with DNAse (Fermentas) and cDNA was then produced utilizing Superscript II (Invitrogen) and random hexamer primers (Invitrogen). qPCR reactions were set up with Maxima SYBR Green/ROX qPCR Master Mix (Fermentas) and the following primers pairs hMyoDF: 5′-CACTCCGGTCCCAAATGTAG-3′ and hMyoDR: 5′-GGTATAAACGTACAAATTCCCTGTA-3′. hMyogeninF: 5′-CAGCGAATGCAGCTCTCAC-3′ and hMyogeninR: 5′-CAGAAGTAGTGGCATCTGTGG-3′. β-actinF: 5′-TTTGAGACCTTCAACACCCCAGCC-3′ and β-actinR: 5′-AATGTCACGCACGATTTCCCGC-3′. Gene expression was quantified using a 7900HT Fast Real-Time PCR System and the 7000 SDS absolute quantification software (Applied Biosystems).

### Chromatin Immunoprecipitation

Heterokaryons were generated as described above. At daily intervals following fusion, cells were fixed in culture medium containing 1% formaldehyde for ten minutes at room temperature. Cross-linking was stopped by addition of glycine to a final concentration of 0.125 M and incubation for five minutes at room temperature. Cells were washed twice with ice cold PBS and harvested by scraping. Pellets were resuspended in 200 µL of lysis buffer (1% SDS, 50 mM Tris-HCl pH 8.0, 10 mM EDTA, containing protease inhibitors (Roche)) and frozen at −80°C. Following collection of all samples, lysates were thawed and sonicated for 25 cycles (20 s ON (high power), 30 s off) using a Bioruptor_300_ (Diagenode). Samples were then diluted 10-fold in dilution buffer (0.01%SDS, 20 mM Tris-HCl pH 8.0, 167 mM NaCl, 1.2 mM EDTA, 1.1% Triton X-100) containing protease inhibitors and were subsequently precleared by incubation with Protein-A beads (Millipore) for 2 hours at 4°C. Ten percent of each lysate was then removed to serve as input samples. The remaining volume was split in half and incubated with either anti-acetyl-histone H3 (Millipore) or normal rabbit IgG (Millipore) overnight at 4°C. Protein-A beads were then added and samples were incubated for 4 hours at 4°C. Antibody-bead complexes were pelleted at 800xg and washed once in TSE I (0.1%SDS, 2 mM EDTA, 20 mM Tris-HCl pH 8.0, 150 mM NaCl, 1% Triton X-100), once in TSE II (0.1%SDS, 2 mM EDTA, 20 mM Tris-HCl pH 8.0, 500 mM NaCl, 1% Triton X-100), once in TSE III (0.25 M LiCl, 1% Deoxycholate, 10 mM Tris-HCl pH 8.0, 1 mM EDTA, 1% NP40) and twice in TE. Samples were resuspended in elution buffer (1%SDS, 0.1 M NaHCO_3_) and incubated for fifteen minutes at room temperature followed by centrifugation at 800xg to pellet the beads. Supernatants were transferred to new tubes and NaCl was added to all samples, including input fractions, to a final concentration of 0.3 M. All samples were then incubated overnight at 65°C to reverse crosslinks. DNA was purified using a QIAquick column (Qiagen) and PCR was performed using primers specific for the human MyoD locus: hMyoDCHIP1F: 5′-CCTGGGCTCCGGGGCGTTTAG-3′ and hMyoDCHIP2R: 5′-GCGCGGCACGGTCCTGGCTT-3′. All data was quantified using a 7900HT Fast Real-Time PCR System and the 7000 SDS absolute quantification software (Applied Biosystems).

### *In vivo* fusion assay

MEF_ Hα7/F_ and MEF_ Hα7_ cells were maintained in DMEM supplemented with 10% fetal bovine serum and 30 µM β-mercaptoethanol. Prior to transplantation, cells were trypsinized, resuspended in PBS and counted. 1×10^5^ cells from each population were then intramuscularly injected into 7-week-old male C57BL/6 recipients utilizing a 26-gauge needle. In the damage model, cells were co-injected with 10 µL of notexin (1 µg/mL) (Latoxan). Muscle tissue was then allowed to heal for one week prior to analysis. As a positive control for the detection of GFP-positive cells in other organs, 1×10^7^ whole bone marrow cells from a GFP-positive mouse (C57BL/6; GFP/CD45.2) were injected intravenously into wild type mice and these recipients were harvested three hours later. For analysis, mice were first terminally anesthetized with avertin, then perfused with PBS containing 10 mM EDTA and finally perfused with 4% PFA in PBS. All lower leg muscles as well as the spleen, lung and liver were then removed from recipients and post-fixed in 4% PFA overnight prior to overnight cryoprotection in 20% sucrose. All tissues were then embedded (OCT, Sakura) and cut into 20 µm (muscle) 10 µm (lung) or 5 µm (spleen and liver) sections (Leica CM3050S). Muscle sections were stained with rabbit anti-mouse laminin (Abcam) overnight at 4°C, followed by a one hour incubation with goat anti-rabbit Alexa 568 (Molecular Probes) at room temperature. Stained muscle tissues were analyzed by confocal microscopy using a Nikon C1 laser scanning confocal microscope and images are presented as maximum intensity projections of *Z* stacks of individual optical sections. For analysis of the spleen, lung and liver, at least 25 sections of each tissue were examined from each recipient (n = 6).

## Supporting Information

Figure S1 **Reprogramming of human NCAM expression following Hα7-mediated fusion** ***in vitro*** **.** (**A**) Human NCAM was expressed by the majority of heterokaryons eight days after Hα7-mediated fusion of MRC-5 cells and differentiating C2C12 myoblasts. (**B,C**) Negative controls demonstrating the lack of human NCAM expression in isolated MRC-5 cells (**B**) and differentiated C2C12 cells (**C**). (**D**) NCAM-positive heterokaryon at eight days post-fusion. Scale bar, 50 µm.
(PDF)

Figure S2 **Absence of MEF_Hα7/F_** **cells in the spleen, lung or liver of transplanted mice.** (**A,C,E**) Positive control demonstrating GFP-positive cells in the spleen (**A**), lung (**C**) and liver (**E**) of a wild type recipient following short term homing of transplanted GFP-positive bone marrow. (**B,D,F**) No GFP-positive cells were observed in the spleen (**B**), lung (**D**) or liver (**F**) of wild type recipients following transplantation of MEF_Hα7/F_ cells. Scale bar, 100 µm.
(PDF)

Table S1 **Percentage of myotubes expressing GFP following Hα7-mediated fusion, PEG-mediated fusion or co-culture of 293T_GFP_ cells and differentiating C2C12 myotubes.**
(PDF)

Table S2 **Fold increase in the number of GFP-positive myotubes observed following Hα7-mediated fusion of 293T_GFP_ cells and differentiating C212 myotubes as compared to the number observed following PEG-mediated fusion.**
(PDF)

Table S3 **Fold increase in human MyoD and myogenin transcript levels detected following Hα7-mediated fusion of MRC-5 cells and differentiating C2C12 myotubes as compared to the levels detected following PEG-mediated fusion.** (Hα7 signal/PEG signal).
(PDF)
