# Supplementary figures and images for: Targeted Cell Fusion Facilitates Stable Heterokaryon Generation In Vitro and In Vivo
Source: PLoS One. 2011 Oct 24;6(10):e26381. doi: 10.1371/journal.pone.0026381 (PMC3200330; doi:10.1371/journal.pone.0026381)

**A**

| Expt #  | NCAM+ Heterokaryons |
|---------|---------------------|
| 1       | 28/33 (85%)         |
| 2       | 25/33 (76%)         |
| 3       | 31/33 (94%)         |
| Average | 85% +/- 9%          |

**B**

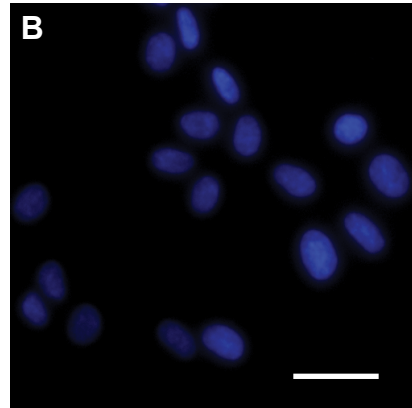

**C**

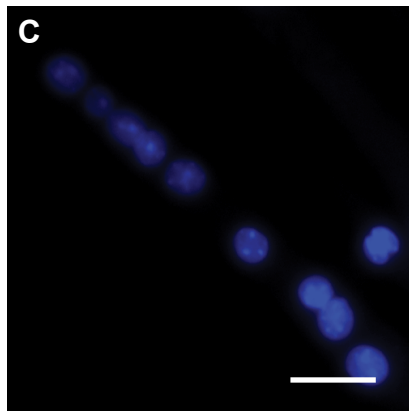

**D**

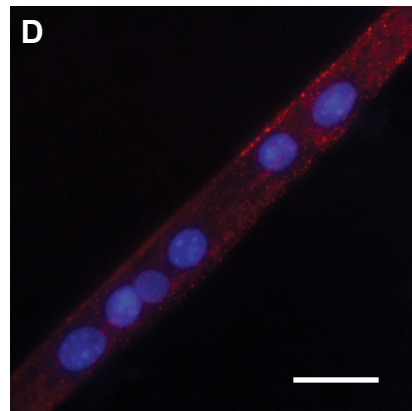

Supplement: Figure S1 — Reprogramming of human NCAM expression following Hα7-mediated fusion in vitro . (A) Human NCAM was expressed by the majority of heterokaryons eight days after Hα7-mediated fusion of MRC-5 cells and differentiating C2C12 myoblasts. (B,C) Negative controls demonstrating the lack of human NCAM expression in isolated MRC-5 cells (B) and differentiated C2C12 cells (C). (D) NCAM-positive heterokaryon at eight days post-fusion. Scale bar, 50 µm. (PDF) [file pone.0026381.s001.pdf]

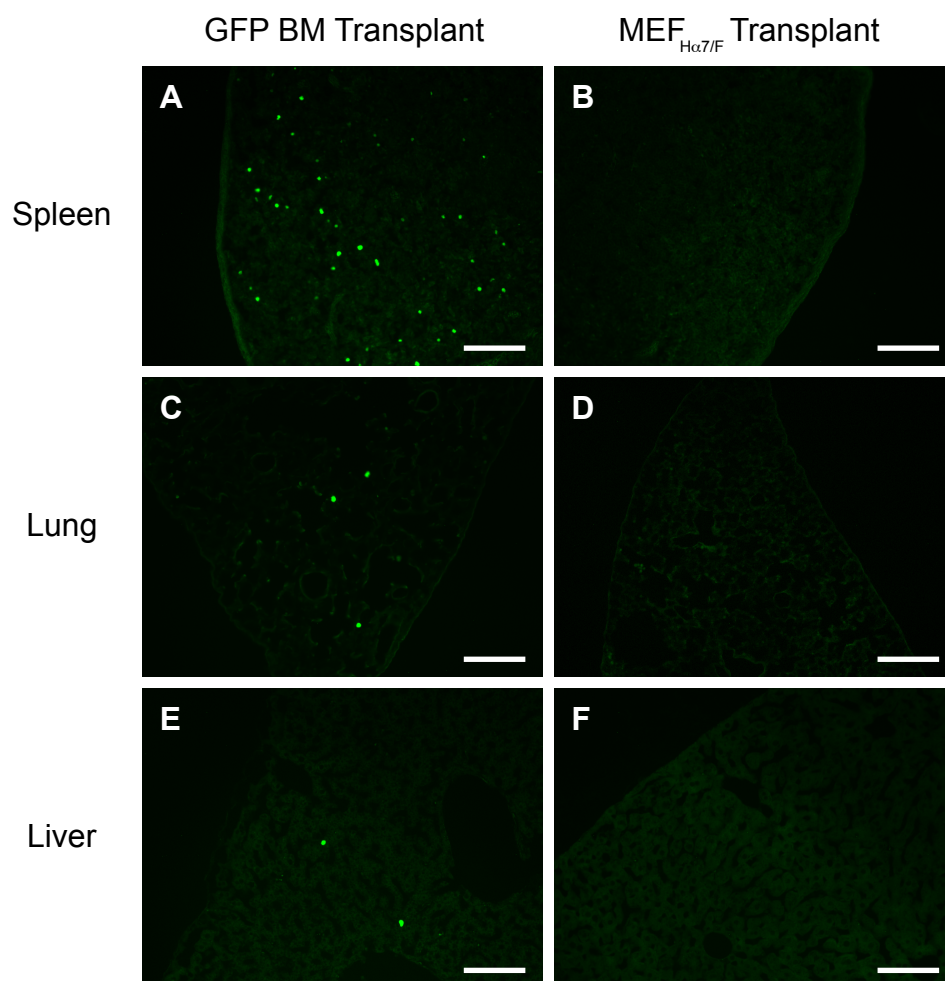

Supplement: Figure S2 — Absence of MEFHα7/F cells in the spleen, lung or liver of transplanted mice. (A,C,E) Positive control demonstrating GFP-positive cells in the spleen (A), lung (C) and liver (E) of a wild type recipient following short term homing of transplanted GFP-positive bone marrow. (B,D,F) No GFP-positive cells were observed in the spleen (B), lung (D) or liver (F) of wild type recipients following transplantation of MEFHα7/F cells. Scale bar, 100 µm. (PDF) [file pone.0026381.s002.pdf]
