# Supplementary material for: Targeted Cell Fusion Facilitates Stable Heterokaryon Generation In Vitro and In Vivo
Source: PLoS One. 2011 Oct 24;6(10):e26381. doi: 10.1371/journal.pone.0026381 (PMC3200330; doi:10.1371/journal.pone.0026381)
Supplement: Table S1 — Percentage of myotubes expressing GFP following Hα7-mediated fusion, PEG-mediated fusion or co-culture of 293TGFP cells and differentiating C2C12 myotubes. (PDF) [file pone.0026381.s003.pdf]

Table S1. Percentage of myotubes expressing GFP following Hα7-mediated fusion, PEG-mediated fusion or coculture of $293T_{GFP}$ cells and differentiating C2C12 myotubes.

Fusogen	Days Post Fusion		
	Day 1	Day 2	Day 3
Hα7	84 ± 14	96 ± 12	92 ± 11
PEG	13 ± 5	11 ± 4	11 ± 7
No Fusogen	0 ± 0	1 ± 1	2 ± 1
