# Supplementary material for: Targeted Cell Fusion Facilitates Stable Heterokaryon Generation In Vitro and In Vivo
Source: PLoS One. 2011 Oct 24;6(10):e26381. doi: 10.1371/journal.pone.0026381 (PMC3200330; doi:10.1371/journal.pone.0026381)
Supplement: Table S2 — Fold increase in the number of GFP-positive myotubes observed following Hα7-mediated fusion of 293TGFP cells and differentiating C212 myotubes as compared to the number observed following PEG-mediated fusion. (PDF) [file pone.0026381.s004.pdf]

Table S2. Fold increase in the number of GFP-positive myotubes observed following Hα7-mediated fusion of $293T_{GFP}$ cells and differentiating C2C12 myotubes as compared to the number observed following PEG-mediated fusion.

Days Post Fusion		
Day 1	Day 2	Day 3
12 ± 4	17 ± 6	16 ± 10
