# Supplementary material for: Targeted Cell Fusion Facilitates Stable Heterokaryon Generation In Vitro and In Vivo
Source: PLoS One. 2011 Oct 24;6(10):e26381. doi: 10.1371/journal.pone.0026381 (PMC3200330; doi:10.1371/journal.pone.0026381)
Supplement: Table S3 — Fold increase in human MyoD and myogenin transcript levels detected following Hα7-mediated fusion of MRC-5 cells and differentiating C2C12 myotubes as compared to the levels detected following PEG-mediated fusion. (Hα7 signal/PEG signal). (PDF) [file pone.0026381.s005.pdf]

Table S3. Fold increase in human MyoD and myogenin transcript levels detected following Hα7-mediated fusion of MRC-5 cells and differentiating C2C12 myotubes as compared to the levels detected following PEG-mediated fusion. (Hα7 signal/PEG signal)

	Days Post Fusion							
	Day 1	Day 2	Day 3	Day 4	Day 5	Day 6	Day 7	Day 8
MyoD	NA[a]	43 ± 28	83 ± 27	94 ± 17	52 ± 12	32 ± 3	4 ± 2	9 ± 1
Myogenin	31 ± 13	11 ± 5	23 ± 16	12 ± 5	6 ± 2	12 ± 5	2 ± 1	9 ± 4

[a] No hMyoD signal was detected on Day 1 following PEG mediated fusion
